# Quetiapine: off-label prescribing in a community mental health team

**DOI:** 10.1192/bjo.2021.212

**Published:** 2021-06-18

**Authors:** Ala Abdelgadir, Richard Walsh, Elizabeth Walsh, Sonn Patel

**Affiliations:** 1 University Hospital Galway; 2 School of Medicine, University College Dublin

## Abstract

**Aims:**

Quetiapine is an atypical anti-psychotic medication licensed for the treatment of schizophrenia, bipolar disorder and adjunctive use in major depressive disorder. It's off-label use in low doses is increasing, possibly due to its sedative qualities, tolerability, low risk of extrapyramidal symptoms and to limit the unnecessary use of benzodiazepines. However, previous research highlights the risk of metabolic consequences even in low doses. Our aim is to establish the prescribing patterns and off-label use of quetiapine within a complete comminity mental health team population (CMHT).

**Method:**

The GR1 CMHT provides care to a population of 25,000 people in a mixed urban and rural area. Multi-disciplinary case notes for all registered patients were reviewed for a one-year period. A database was created to include sociodemographic details, diagnosis, and medication. The proportion of patients prescribed quetiapine was identified and the dosage divided into multiple increments. The team's consultant reviewed and verified all ICD-10 diagnoses. Quetiapine dose by diagnosis was examined using descriptive statistics.

**Result:**

Of 246 registered patients, 62 (25% of CMHT caseload) were prescribed Quetiapine. Quetiapine was prescribed across a range of disorders including psychotic 17 (27%), mood 18 (29%), anxiety 14 (22 %), personality disorders 11 (18%) and others 2 (3%). Doses spanned between 25 mg – 800 mg daily. 19 patients (31%) were prescribed less than 25 mg, 20 patients (32%) between 25 mg and 100 mg and 23 patients (37%) above 100 mg. In psychotic and mood disorders, dosage varied widely between the low and high range. Furthermore, of the psychotic disorders, 11 (65%) were prescribed a second antipsychotic medication. For diagnoses in which the prescribing indication was clearly off-label, the dosages were predominantly low (100 mg or less).

**Conclusion:**

Quetiapine was commonly prescribed in our patient population. Its frequent off-label use in low doses suggests that its prescription was for its additional qualities. Our findings highlight the importance of assessing the risk-benefit profile for every patient given the potential side effects, involving patients in the consultation of its off-label use and appropriate monitoring.

